# A Comprehensive Review of Non-Steroidal Anti-Inflammatory Drug Use in The Elderly

**DOI:** 10.14336/AD.2017.0306

**Published:** 2018-02-01

**Authors:** Supakanya Wongrakpanich, Amaraporn Wongrakpanich, Katie Melhado, Janani Rangaswami

**Affiliations:** ^1^Department of Medicine, Einstein Medical Center, Philadelphia, Pennsylvania, PA 19141, USA.; ^2^Department of Pharmacy, Mahidol University, Bangkok 10400, Thailand; ^3^Division of Nephrology, Department of Medicine, Einstein Medical Center, Philadelphia, PA 19144, USA

**Keywords:** Non-steroidal anti-inflammatory drug, NSAIDs, elderly, geriatric conditions, pleiotropic effects

## Abstract

NSAIDs, non-steroidal anti-inflammatory drugs, are one of the most commonly prescribed pain medications. It is a highly effective drug class for pain and inflammation; however, NSAIDs are known for multiple adverse effects, including gastrointestinal bleeding, cardiovascular side effects, and NSAID induced nephrotoxicity. As our society ages, it is crucial to have comprehensive knowledge of this class of medication in the elderly population. Therefore, we reviewed the pharmacodynamics and pharmacokinetics, current guidelines for NSAIDs use, adverse effect profile, and drug interaction of NSAIDs and commonly used medications in the elderly.

NSAIDs are one of the most commonly prescribed classes of medication for pain and inflammation [1]. They are responsible for approximately 5-10% of all medications prescribed each year [2]. The prevalence of NSAID use in patients over 65 years old is as high as 96% in the general practice setting [3]. Approximately 7.3% of elderly patients over 60 years old filled at least one NSAID prescription in one year period [4]. In addition to their anti-inflammatory effect, NSAIDs have antipyrexic and analgesic properties. These medications inhibit Cyclooxygenases (COXs) enzymes, which are rate-determining enzymes for prostaglandins and other prostanoids synthesis, such as thromboxanes.

Compared with Nonselective NSAIDs that inhibit both COX-1 and COX-2, COX-2 inhibitors (as known as coxibs) inhibit only COX-2 enzymes. COX-2 plays more of a role in prostaglandin mediated pain and inflammation, while COX-1 plays some housekeeping role in the protection of gastric mucosa and in platelet hemostasis. While the gastro intestinal safety profiles of COX-2 inhibitors have improved, the cardio-nephrotoxic adverse effects are still significant [5].

Several known adverse effects of NSAIDs in the elderly have been studied (Table 1), however, recent literature reveals that there might be beneficial roles of the anti-inflammatory effect of NSAIDs in targets such as improvement in cognitive function.

## Pharmacodynamics and Pharmacokinetics of NSAIDs

The major therapeutic actions of NSAIDs are primarily enacted by their ability to block certain prostaglandins (PGs) synthesis through the cyclooxygenase enzymes (COX-1 and COX-2) inhibition. COX-1 produces prostaglandins and thromboxane A2 which control mucosal barrier in GI-tract, renal homeostasis, platelet aggregation and other physiological functions. COX-2 produces PGs that related to inflammation, pain and fever. COX-1 is expressed in normal cells, while COX-2 is induced in inflammatory cells [6-8]. COX-2 inhibition most likely represents the desired effect of NSAIDs’ anti-inflammatory, antipyretic and analgesic response; while COX-1 inhibition plays a major role in the undesired side effects such as GI and renal toxicities.

**Table 1:** NSAIDs’ common adverse effect profile.

Gastrointestinal toxicity	•Dyspepsia •Gastroduodenal ulcers •GI bleeding and perforation
Cardiovascular adverse effects	•Edema •Hypertension •Congestive heart failure •Myocardial infarction •Stroke and other Thrombotic events
Nephrotoxicity	•Electrolyte imbalance •Sodium retention •Edema •Reduce glomerular filtration rate •Nephrotic syndrome •Acute interstitial nephritis •Renal papillary necrosis •Chronic kidney disease

Most NSAIDs are well absorbed in the gastrointestinal tract and have high bioavailability. Some drugs such as diclofenac undergo hepatic first-pass metabolism which resulted in the reduction in bioavailability. While some drugs such as sulindac and parecoxib are prodrugs and need hepatic metabolism to become their active metabolites (sulindac sulfide and valdecoxib, respectively). NSAIDs are highly bound to plasma proteins. NSAIDs are usually metabolized in the liver and excreted in the urine. Common NSAIDs drug have a variable half-life; they can be anywhere from 0.25-0.3 hours such as aspirin or 45-50 hours such as piroxicam [9, 10]. All these pharmacokinetics parameters can change with aging since the elderly have low body water compared with adults. Protein binding may be reduced and volumes of distribution may be altered.

## Current guideline and the use of NSAIDs

In 1986, The World Health Organization (WHO) developed the analgesic ladder for the treatment of cancer pain with the three-step sequential approach for pain medication administration depending on the severity of pain. NSAIDs are considered group one medications, recommended for mild pain and are the first step in treating pain [11]. They are commonly prescribed in the setting of acute pain, such as acute musculoskeletal injury. In addition, they are also commonly used in the setting of arthritic pain and exceed the analgesic effects of acetaminophen, because of their anti-inflammatory effects [1, 12].

Professional societies, including American Geriatric Society, American College of Rheumatology, and the European League Against Rheumatism, recommend using NSAIDs with caution and limit their use to the lowest effective dose and shortest duration. They recommend that, when used, common gastrointestinal, renal and cardiovascular side effects should be routinely monitored [1, 13, 14]. Considering this recommendation, the prevalence of inappropriate use of NSAIDs is concerning. In 2015, Ussai et al., did a retrospective study of 3,050 subjects with chronic pain[15]. They found that 97% of chronic pain subjects took NSAIDs for more than 21 consecutive days.

The American Geriatric Society updated the Beers Criteria in 2015. They recommended that the chronic use of all NSAIDs, including high dose aspirin, should be avoided because of the risk of gastrointestinal bleeding. High-risk groups include: age above 75 years, corticosteroid use, current use of anticoagulants or antiplatelet agents [16].

## NSAIDs’ known adverse effects

### NSAIDs and Kidneys

Compared with GI and cardiovascular risks, Renal side effects of NSAIDs are considered uncommon. However, advanced age puts patients at higher risk of developing nephrotoxicity from NSAIDs. NSAIDs cause inhibition of prostaglandin and thromboxane synthesis leading to renal vasoconstriction and consequently reduced renal perfusion and aberrant renal function. Clinical manifestations of NSAID induced nephrotoxicity includes electrolyte imbalance such as hyperkalemia, reduce glomerular filtration rate, nephrotic syndrome related to drug induced minimal change disease, chronic kidney disease, acute interstitial nephritis, sodium retention, edema, and renal papillary necrosis [5].

It is possible that each individual and type of NSAIDs play an important role in AKI development. Ungprasert et al. recently published an elegant systematic review and meta-analysis of observational studies regarding NSAIDs and risk of AKI. They found a statistically significant elevated AKI risk among traditional NSAIDs users. The pooled risk ratios of specific COX-2 inhibitors and the two traditional NSAIDs with the most COX-2 selectivity (diclofenac and meloxicam) were also comparable with other traditional NSAIDs even though they did not achieve a statistical significant [16].

Gooch et al. studied effects of NSAID use on the progression of CKD in elderly subjects over the age of 66, in a prospective community-based study with a sample size of over 10,000 subjects [17]. They concluded that high cumulative NSAIDs exposure is associated with an increased risk for rapid CKD progression. Regarding acute kidney injury (AKI) associated with NSAIDs use in the elderly, Kate et al. built a prediction model for AKI in hospitalized older adults. They found that medication combinations such as NSAIDs and diuretics can predict acute kidney injury [18].

The American Geriatric Society (AGS) recommends that all NSAIDs should be avoided in patients with stage IV and V CKD (creatinine clearance less than 30 mL/min) [19, 20].

### NSAIDs and Gastrointestinal (GI) adverse effects

Aging itself can increase risk of GI bleeding [21]. It is known that GI bleeding and ulceration from NSAIDs use increase in severity and frequency with increasing age [1]. NSAID use increases the risk of GI bleeding in the elderly four folds [21]. The mechanism underlying NSAIDs induced GI adverse effects lies in the fact that these medications inhibit prostaglandin synthesis, causing weakening of the protective GI mucosal barrier, predisposing one to bleeding.

NSAIDs-induced gastroduodenal ulcers can be prevented by the use of GI protective agents, such as, Misoprostol, H2-receptor antagonists (H2RA) or proton pump inhibitors (PPI)[22]. This strategy is used in approximately 20% of elderly patients who are on chronic NSAIDs [4]. Another strategy to minimize GI adverse effects is to substitute nonselective NSAIDs with COX-2 selective NSAIDs. Multiple studies have revealed that COX-2 inhibitors, such as lumiracoxib, celecoxib, and rofecoxib, caused less damage to GI mucosa compared to non-selective NSAIDs [23-25]. Rhame et al. confirmed these findings when they studied elderly patients on low-dose aspirin. They found that celecoxib has superior GI safety profile, compared with non-selective NSAIDs [26].

However, there are increase risks of cardiovascular adverse effects with the use of COX-2 inhibitors [27]. Thus, tailoring a patient’s GI risk factors versus cardiovascular risk factors is necessary to determine the choice of GI protection options for patients on chronic NSAIDs [22].

### NSAIDs and cardiovascular adverse effects

Since rofecoxib and valdecoxib, were withdrawn from market in 2004 and 2005 respectively, due to adverse cardiovascular events such as edema, myocardial infarction, thrombotic events, stroke and hypertension, concerns regarding all COX-2 inhibitors potential for cardiovascular adverse effects have been raised [27-30].

Page et al. conducted a case-control study in elderly patients who were first hospitalized with congestive heart failure. They compared NSAIDs user (all NSAIDs other than low dose aspirin) and non- user. The use of NSAIDs was associated with increased risk of first hospital admission due to congestive heart failure (OR 2.1, 95% CI 1.2-3.3)[31].

Thus, all NSAIDs (COX-2 and non-selective) may be associated with increased cardiovascular adverse effects and each medications’ risk/benefit profile should be considered before prescribing to individual patients [26].

### NSAIDs and Blood Pressure

In almost 60% of elderly patients, NSAIDs were co-prescribed with medications for hypertension and/or congestive heart failure [4]. Non-selective NSAIDs are known to attenuate the antihypertensive effect of some specific blood pressure medications, such as ACE inhibitors. However, no similar effect was observed with COX-2 inhibitors [32]. In general, NSAIDs can increase blood pressure by 5 mmHg in average. The mechanism of NSAIDs promoting hypertension is hypothesized to be related to the inhibition of prostaglandin synthesis, which leads to an interference of renal vasculature which manipulates the regulation of blood pressure. In addition, NSAIDs themselves can cause elevation of serum aldosterone, leading to sodium retention and hypertension [33].

Johnson et al. performed a study of 2,805 community dwelling people over the age of 60, and found the prevalence of NSAID use to be 26%. They reported that NSAID use can predict the presence of hypertension with an odds ratio of 1.4 (95% CI 1.1-1.7)[34].

## NSAIDs and common geriatric conditions

### Dementia and cognitive decline

Alzheimer’s disease is the most common form of dementia. There is evidence of inflammation in Alzheimer’s brain in vivo and in vitro[35]. In 2003, Kang et al. published results from telephone surveys of 16,128 participants from The Nurses’ Health Study Cohort. They found that long-term NSAIDs users showed reduced odds of impaired cognitive function [36]. Later, a prospective study among 4,409 elderly individuals by Grodstein et al. revealed that the long-term use of ibuprofen was associated with slower rates of cognitive decline [37].

Ancelin et al. conducted a 7-year prospective study to investigate the effect of NSAIDs on cognitive function in elderly patients older than 65 years [38]. They found no significant association with either dementia or cognitive decline incidence. Similar findings were found in a population based epidemiological study of 2,422 subjects by Wichmann et al. in which NSAIDs use has no association with incident cognitive impairment or dementia rates [38].

In preexisting dementia, COX-2 inhibitors did not show any benefit of slowing progression of cognitive diseases. Soininen et al. did a multicenter randomized controlled trial over 1-year period and they found no association between celecoxib use (200 mg twice a day) and Alzheimer’s disease progression [39].

### Depression

Evidence suggests that elevation of pro-inflammatory cytokines is linked to major depression; NSAIDs were believed to play some role in mitigating the anti-inflammatory effects of depression. In adults over 18 years of age, NSAIDs had a statistically significant antidepressant effect in bipolar depression [40], however, this effect was not seen in the elderly with depression. Fields et al. evaluated the effects of celecoxib and naproxen on depressive symptoms in elderly people over the age of 70 in a randomized controlled trial and found no association between treatment groups compared to placebo in terms of late life depression [41].

### Musculoskeletal effects

In 2015, Jankowsky et al. conducted an RCT of 189 elderly subjects between 60 and 75 years of age [42]. They determined the effects of ibuprofen use and bone mineral density (BMD) adaptations after 36 weeks of exercise. They found no difference between treatment groups and placebo.

Beyer *et al.* conducted a double blind randomized controlled trial using piroxicam versus placebo in geriatric patients over the age of 70 who were hospitalized with infection-induced inflammation (characterized by C-reactive protein (CRP) serum level > 10 mg/L and/or fibrinogen > 400 mg/dL)[43]. They discovered that piroxicam improved muscle performance compared with placebo. Thus, NSAIDs may play essential role in reducing infection-induced inflammation in this particular scenario.

Regarding falls, which can lead to morbidity and mortality in the elderly, NSAIDs seem to be a significant risk factor. Data from Walker et al. suggested that NSAIDs (including low-dose aspirin) increase a likelihood of falling by 10 folds [44]. A meta-analysis by Woolcott et al. of multiple medication classes related to falls in the elderly revealed an unadjusted odds ratio for falls with NSAID use to be 1.21 (95% CI, 1.01-1.44)[45].

Polypharmacy is an important factor that increases the risk of falls in the elderly [46-48]. Interestingly, a recent publication from Zia et al. in 2016 revealed that the use of two or more fall risk-increasing drug (FRIDs), but not polypharmacy per se, was a significant predictor for falls [49]. NSAIDs are considered an important group of FRIDs. This finding suggests that falls associated with polypharmacy in previous studies may be due to the use of multiple FRIDs.

## Urinary incontinence

In animal models, NSAIDs improved bladder function and decrease micturition frequency[50]. In humans, Saito et al. investigated the effectiveness of loxoprofen sodium in the management of nocturia in benign prostatic hyperplasia and overactive bladder in elderly patients. They found significant improvement in term of frequency and volume of nocturia [51]. This finding highlights the treatment benefits of NSAIDs in overactive bladder.

### Psychiatric events

NSAIDs-associated psychiatric events are less common, but still relevant in clinical practice. In 2004, Onder et al. reviewed medical literature regarding NSAIDs and psychiatric events and found 453 cases reported. In their findings, most patients were elderly. Psychiatric symptoms included psychosis, agitation, depression, anxiety, paranoia, delirium, mania, and hallucinations [2]. An exact mechanism of NSAIDs-associated psychiatric symptoms is unknown but believed to be involved in altering prostaglandins and prostaglandin precursors in the central nervous system.

### Cancer risk

There is a growing body of evidence that NSAIDs are associated with decrease risk of various types of cancer, such as, endometrial cancer [52], esophageal, head and neck cancer [53], and prostate cancer [54]. The postulated mechanism of NSAIDs and cancer risk reduction has been explained by several mechanisms: 1) NSAIDs inhibit COX-2 expression, which plays major role in tumor initiation, tumor progression, and suppression of antineoplastic immune cells 2) direct effect of NSAIDs in inhibiting cancer cells proliferation and apoptosis induction [31].

However, in cervical cancer [55], there is no association between NSAIDs and cancer risk. In the Women’s Health Initiative (WHI), that followed 129,013 participants over 9.7 years, chronic and consistent NSAIDs use was not associated with reduction of total cancer risk (HR 1.00, 95% CI: 0.94-1.06). However, in some specific types of cancer including, colorectal cancer, ovarian cancer, and melanoma, NSAIDs were associated with reduced risks [56].

NSAIDs may play some role in palliation for advanced stage cancer. NSAIDs combined with megestrol acetate increased weight and improved quality of life in advanced gastrointestinal cancer patients compared with megestrol acetate alone [57]. Apart from cancer cachexia, NSAIDs may improve physical performance and self-reported quality of life in cancer patients [58].

### Stroke

NSAIDs can increase the risk of stroke, and the risk varies with different types of NSAIDs. A population-based case-control study by Garica-Posa et al. revealed that certain NSAIDs including diclofenac (OR = 1.53; 95% CI, 1.19-1.97) and aceclofenac (OR = 1.67; 1.05-2.67) increase the risk of stroke. However, they found no association with naproxen or ibuprofen [59]. In 2011, Roumie et al. conducted a meta-analysis to investigate the cerebrovascular risk of NSAIDs [30]. They found insufficient evidence to confirm any NSAID to be safe in terms of cerebrovascular risk profile. Compared with naproxen, the least harmful NSAID for cardiovascular outcomes, valdecoxib was associated with the highest risk of stroke (adjusted HR 1.41, 95% CI 1.04, 1.91).

**Table 2:** Drug interaction of NSAIDs and commonly used medications.

Medication	Interactions
Antiplatelets (aspirin, clopidogrel)	Increases risk of GI bleeding
Angiotensin-converting-enzyme inhibitor (ACEI) and Angiotensin Receptor Blockers (ARB)	Increases in blood pressure by attenuating antihypertensive effects
Beta blockers	Increases in blood pressure by attenuating antihypertensive effects
Calcium antagonists	Increases in blood pressure by attenuating antihypertensive effects
Corticosteroids	Increases risk of GI bleeding
Digitalis glycosides	Increase serum digoxin level
Diuretics	Increases in blood pressure by attenuating antihypertensive effects
Methotrexate	NSAIDs reduce renal excretion of methotrexate, causing methotrexate toxicity.
Selective serotonin reuptake inhibitors (SSRIs)	Increases risk of GI bleeding
Warfarin and other anticoagulants	Increases risk of GI bleeding

In 2011, Barthelemy et al. studied an impact of NSAIDs on cardiovascular outcome including stroke. They found that long-term use of all NSAIDs increases risk of stroke by 64% at two years[60]. There are several potential mechanisms of NSAIDs associated with stroke. NSAIDs can interfere with vasoconstriction and sodium excretion causing hypertension, a major risk factor of stroke. Also, these medication can induce platelet aggregation and increase thrombus formation [61].

In a post-ischemic stroke setting, it is believed that non-specific inflammation limits neurorecovery. Thus, NSAIDs may play some roles in reducing inflammation after an ischemic stroke. Sandu et al. studied the benefits of indomethacin after acute cerebral ischemia in a rat model of stroke [62]. They found several positive consequences, such as an increase in the number of surviving neurons and a decrease in infarct size, however, this effects was reduced in the aged rat.

## Drug Interaction

NSAIDs are one of the most common causes of adverse drug reactions [63]. As patient age, and the number of medications increase, NSAIDs in the elderly should be prescribed with caution. NSAIDs concomitantly used with specific medication can alter the risk of gastrointestinal ulceration and/or bleeding (Table 2). These drugs include selective serotonin reuptake inhibitors (SSRIs), corticosteroids, digitalis glycosides, diuretics, beta blockers, calcium antagonists, angiotensin converting enzyme, warfarin, clopidogrel, aspirin, and other anticoagulants [4, 14, 20, 64]. Some specific NSAIDs were found to reduce renal clearance of methotrexate, a commonly used medication for rheumatoid arthritis [65].

In the primary care setting, Koffeeman et al. conducted a retrospective cohort study using a database of 1.5 million patients in the Netherlands who were prescribed NSAIDs due to musculoskeletal pain symptoms [66]. They found that 6% of all patients consulted their primary care provider due to non-serious potential adverse drug reactions and the most common symptom was dyspepsia (32%).

## Conclusion

In order to provide comprehensive care of the elderly, knowing the mechanism of action, current guidelines, adverse drug reaction, and the pleiotropic effects of common drugs is important. NSAIDs are one of the most commonly prescribed drugs in the elderly. These medications should be prescribed for the shortest duration possible in the lowest effective dose, and with careful surveillance to monitor GI, renal, and cardiovascular toxicity. This is especially true for elderly patients who are very susceptible to the side effect profiles of NSAIDs. There is some evidence to support the role of NSAIDs in dementia prevention, improve muscle performance, improve urinary incontinence, and decrease the risk of some specific cancers. However, NSAIDs can also increase the risk of falls, increase geriatric psychiatric events, and increase the risk of stroke. Thus, these risks and benefits should be balanced carefully in individual patients to optimize overall outcomes, especially in the elderly.
